# Clerical Mortuary

**Published:** 1859-09

**Authors:** 


### CLERICAL MORTUARY.

THE lowest certainly ascertained death rate in the world, is fifteen annually out of every thousand; the highest is more than double, and is found in the most notorious districts in England, being thirty-six out of every thousand.

Of the twenty-five hundred ministers belonging to the Old School Presbyterian body in May, 1858, thirty-one died within the year following, making their death rate twelve and a half, or one-sixth lower than the most favored people known on the earth, as to health.

There is not in this wide Union of States a better educated, a more active, energetic, and indomitable class of workers than the Presbyterian clergy, while their average salaries are less than almost any others known, less than that of Methodist preachers. The ligitimate inference is, that piety, a high grade of mental culture, a steady activity in the discharge of ministerial duties, combine to produce the greatest ascertained exemption from death on the globe: and it cannot be doubted by the reasonable and unprejudiced, that if they were more liberally paid, and thus had more leisure for the prosecution of their legitimate duties, their death rate would be diminished to a lower figure still, by reason of that exemption from the hurries, the exposures, the over efforts, and the wearing solicitudes which a meagre sustension necessarily imposes on all sensitive, educated, and conscientious minds.

